# Contribution of Atmospheric Diffusion Conditions to the Recent Improvement in Air Quality in China

**DOI:** 10.1038/srep36404

**Published:** 2016-11-02

**Authors:** Xiaoyan Wang, Kaicun Wang, Liangyuan Su

**Affiliations:** 1 Insitude of Atmospheric Science, Fudan University, Shanghai, 200433, China; 2 College of Global Change and Earth System Science, Beijing Normal University, Beijing, 100875, China; 3 Joint Center for Global Change Studies, Beijing, 100875, China

## Abstract

This study analyzed hourly mass concentration observations of PM_2.5_ (particulate matters with diameter less than 2.5 μm) at 512 stations in China from December 2013 to May 2015. We found that the mean concentrations of PM_2.5_ during the winter and spring of 2015 Dec. 2014 to Feb. 2015 and Mar. 2015 to May 2015) decreased by 20% and 14% compared to the previous year, respectively. Hazardous air-quality days decreased by 11% in 2015 winter, with more frequent good to unhealthy days; and the good and moderate air-quality days in 2015 spring increased by 9% corresponding to the less occurrence of unhealthy conditions. We compared the atmospheric diffusion conditions during these two years and quantified its contribution to the improvement of air quality during the first half of 2015 over China. Our results show that during the 2015 winter and spring, 70% and 57% of the 512 stations experienced more favorable atmospheric diffusion conditions compared to those of previous year. Over central and northern China, approximately 40% of the total decrease in PM_2.5_ during the 2015 winter can be attributed to the favorable atmospheric diffusion conditions. The atmospheric diffusion conditions during the spring of 2015 were not as favorable as in winter; and the average contributions of the atmospheric conditions were slight.

PM_2.5_ (particulate matters with diameter less than 2.5 μm) concentrations have attracted considerable attention due to their effects on human health^1^,^2^,^3^,^4^,^5^. In addition, PM_2.5_ can reduce visibility and directly affect land, sea and air traffic safety^6^,^7^,^8^,^9^. The interaction between particulate matter and the climate system should not be overlooked^10^,^11^,^12^,^13^,^14^,^15^. Severe PM_2.5_ pollution episodes have occurred frequently over China in recent years^16^,^17^,^18^,^19^,^20^. Air pollution has become a top concern for Chinese citizens, particularly since the occurrence of several severe pollution episodes in January 2013; as a result, China has “declared war” on pollution^21^,^22^,^23^,^24^,^25^.

In September 2013, the Chinese State Council issued the Action Plan on Prevention and Control of Air Pollution to prevent air pollution with the goal of achieving a 15–20% decrease in PM_2.5_ concentrations over three economic zones in 2017 compared with 2012^26^. New air-quality regulations have been updated, and some local and regional principles for air-quality management have been designed to control and protect air quality. PM_2.5_ and other air pollutants have been measured nationwide on hourly basis since January 2013. A good news is that the air quality in the first half of 2015 was substantially better than that during the same period in 2014. This improvement has been attributed to the reduction of emissions from coal-fired power plants, an increase in non-fossil-fuel energy, reduced vehicle emissions, and new emission standards and environmental laws^27^,^28^,^29^,^30^,^31^.

It is well known that local meteorological conditions can determine the variability of air quality in brief time periods (hourly to daily) by removing and transporting pollutants^21^,^32^,^33^,^34^,^35^,^36^,^37^,^38^. A favorable atmospheric diffusion condition can result in better air quality. However, the national governments assess seasonal or annual air quality just by comparing the air quality data with those in the previous year, and completely ignored the large year-to-year difference of air quality due to the difference of atmospheric diffusion conditions, which will lead to the misunderstanding about the attribution to the change of air quality^39^. Therefore, the goal of this study is to diagnose how much of the improvement of air quality in the first half of 2015 over China can be attributed to the change in atmospheric diffusion conditions.

## Results

### Improved Air Quality in the First Half of 2015

**Figure 1:**
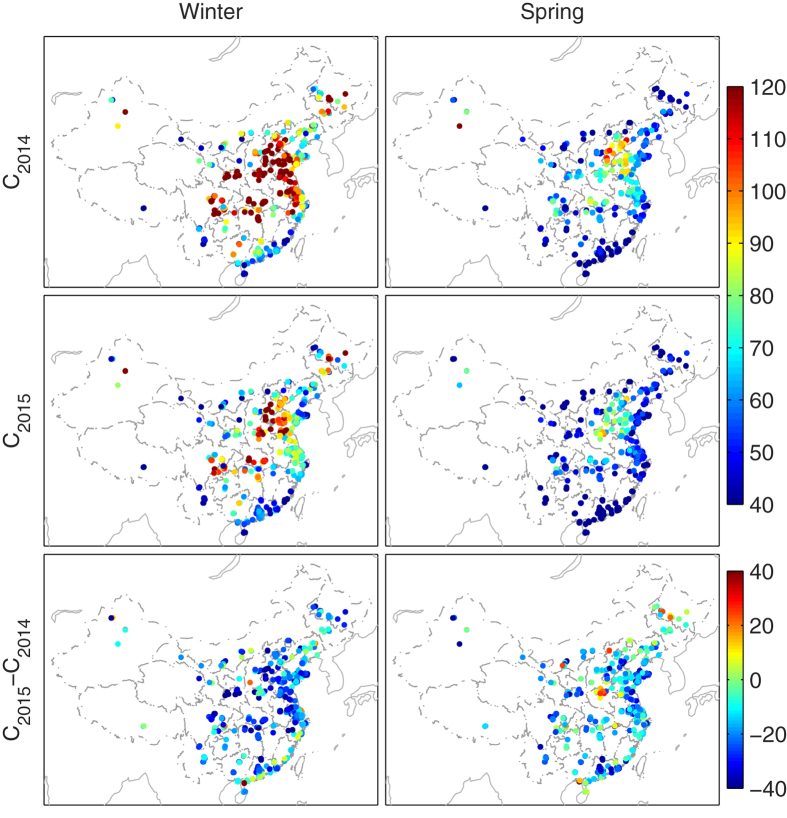
Seasonal mean PM_2.5_ concentrations during winter and spring of 2014 and 2015 (unit: μgm^−3^) and the relative difference in these two years (in the bottom panel, unit: %). A negative difference in the bottom panel indicates a decrease of PM_2.5_ in 2015 compared with that in 2014; 453 of the 512 stations showed a decreased PM_2.5_ in 2015 wintertime, and 445 stations showed a decreased PM_2.5_ in springtime. The statistical results of this figure are summarized in Table 1. This figure was produced by Matlab version 7.13 (http://www.mathworks.com/products).

Seasonal PM_2.5_ mass concentrations were used to evaluate the variation of local air quality. We compared the air quality in the first half of 2015 (i.e. wintertime: Dec. 2014–Feb. 2015 and springtime: Mar. 2015–May 2015) to the same period of 2014. Figure 1 shows the seasonal mean PM_2.5_ mass concentrations during the winters and springs of 2015 and 2014 and their relative differences. A higher seasonal PM_2.5_ concentration occurred at the intersection region of Hebei, Shandong, Shangxi and Henan Provinces, particularly during the 2014 winter, with better air quality in the east coastal area than in the inland regions, and better quality in the spring than in the winter. The PM_2.5_ concentrations decreased considerably in the first half of 2015 compared with the same period of 2014, by 20% and 14% during the winter and spring, respectively (summarized in Table 1). A total of 88.5% stations reported decreased PM_2.5_ concentrations during the winter of 2015, and 86.9% stations for the 2015 spring compared to that of 2014.

**Figure 2:**
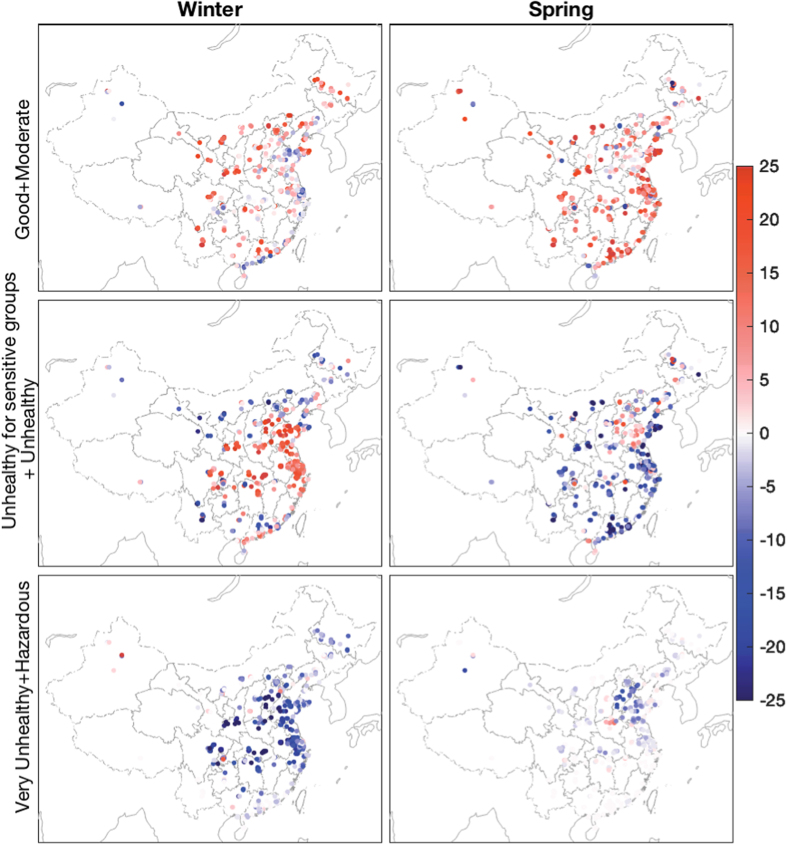
Differences of the occurrence frequency of major air-quality levels between 2014 and 2015 (unit: %). A positive value indicates more frequent occurrence of the specific air quality in 2015 than in 2014. The winter of 2015 showed a substantial decrease in very unhealthy and hazardous air quality, with more frequent good and unhealthy conditions than the previous year, indicating better air quality in 2015. Similarly, the occurrence of good and moderate air quality increased in the spring of 2015 due to the decrease in unhealthy conditions. Stations without specific air-quality levels in both years are not shown. The statistical results of this figure are summarized in Table 2. This figure was produced by Matlab version 7.13 (http://www.mathworks.com/products).

The occurrence of different air-quality levels is shown in Fig. 2. Six categories of air-quality levels (as shown in Table 2 and Table S1) are defined based on the air-quality index (AQI) of the U.S. Environmental Protection Agency^40^; we combined them into three major levels with increasing air pollution conditions: good and moderate, unhealthy for sensitive and unhealthy, and very unhealthy and hazardous group. Compared with the winter of 2014, it showed a substantial decrease in hazardous air quality occurrences in 2015 winter but more frequent unhealthy and good air-quality occurrences, indicating that the decreased seasonal mean PM_2.5_ concentration was primarily due to the infrequency of severely polluted days. Approximately 40% of the stations did not record hazardous air quality in both spring seasons. As summarized in Table 2, good and moderate air-quality occurrences were 11% more frequent in the spring of 2015, corresponding to a 9% decrease in unhealthy condition occurrences.

### Contribution of Atmospheric Diffusion Conditions to the Improved Air Quality

**Figure 3:**
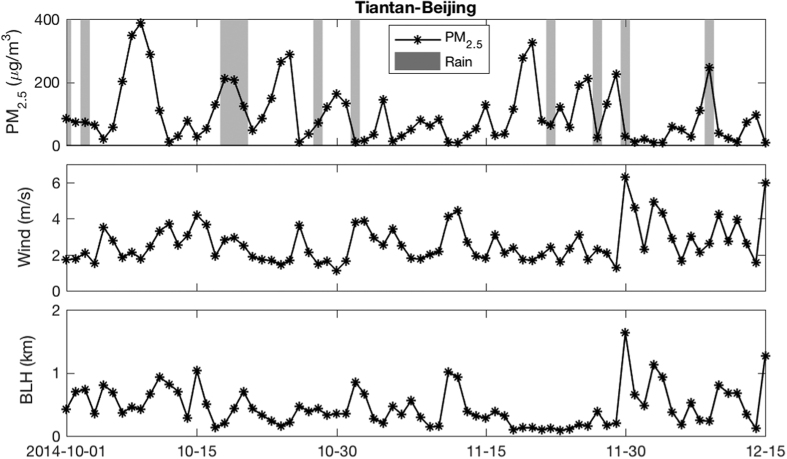
Time series of PM_2.5_ mass concentration, surface wind, boundary layer height and precipitation period from Oct. 1 to Dec. 15, 2014, at Beijing Tiantan station. The shaded area indicates the precipitation period. PM_2.5_ concentration decreased with the increased wind speed and boundary layer height. A significant wet deposition effect was apparent during precipitation events. This figure was produced by Matlab version 7.13 (http://www.mathworks.com/products).

Air pollutants emissions from a particular area do not typically change over a short period, but local meteorological patterns can strongly affect the accumulation of air pollutants via removal and transport. Pollution episodes are usually related to the presence of air stagnation conditions with poor atmospheric horizontal transport or vertical diffusion abilities^21^,^34^,^41^,^42^,^43^,^44^. Another important factor for contaminant scavenging is wet deposition due to precipitation^45^,^46^,^47^. Figure 3 shows the variation of PM_2.5_ and the simultaneous atmospheric conditions at a Beijing station from Oct. 1 to Dec. 15, 2014, during which the PM_2.5_ concentration could dramatically increased from below 10 μgm^−3^ to approximate 400 μgm^−3^. Precipitation always tends toward the deposition of pollutants with a lower PM_2.5_ concentration during rainy days. Sustained weak surface wind and a shallow atmospheric boundary layer can trigger an air pollution episode, which can be broken by heavy surface wind or a well-developed boundary layer.

**Figure 4:**
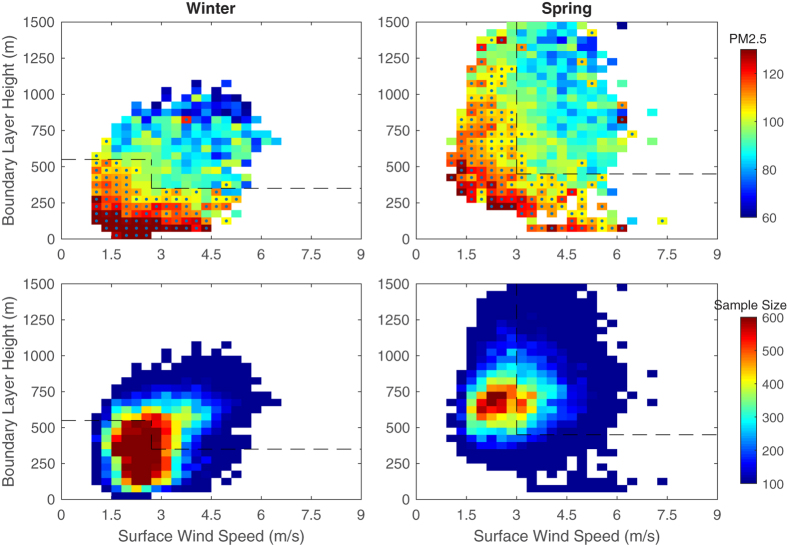
Dependence of normalized daily PM_2.5_ concentrations on surface wind speed and boundary layer height (top panel) and the number of samples in each grid (bottom panel). Datasets from the winter and spring of 2014 and 2015 throughout the entire country (512 stations) were used. Daily PM_2.5_ concentrations were normalized by the monthly mean of the specific year to avoid the effect of spatial and temporal variation. All the precipitation events were excluded in the dependence relationship to rule out the effect of wet deposition. Dot signs in the top panel indicate that the average of the normalized daily PM_2.5_ in this grid is greater than 100%, which means the daily PM_2.5_ concentration exceeded its monthly mean. The lower-left corner of the dashed line shows the condition of air stagnation with a higher PM_2.5_ concentration. Grids with samples exceeding 50 are shown. This figure was produced by Matlab version 7.13 (http://www.mathworks.com/products).

Air stagnation is typically used to describe the atmospheric capability for air pollutant diffusion, which involves horizontal transport, vertical diffusion and wet deposition; however, air stagnation criteria are sensitive to local meteorological conditions, and existing air stagnation definitions are empirical^43^,^46^. Therefore, we proposed a quantitative criterion to identify the occurrence of air stagnation condition based on the daily boundary layer height (BLH), surface wind speed and the present of precipitation (as shown in Fig. 4, more detailed information in Method). Daily surface wind speed and boundary layer height were used to indicate the horizontal and vertical diffusion ability of the atmosphere, and the present of precipitation is considered to be benefit to the wet deposition by default. Figure 4 shows that when there is no effective precipitation, near-surface PM_2.5_ concentrations decreased with the increase of surface wind speed and BLH, i.e., shallow boundary layer and weak surface wind restricts the diffusion of near-surface pollutants. Thus, a day was considered as air stagnation day when the weak surface wind (2.7 ms^−1^ for winter and 3 ms^−1^ for spring) companied with shallow BLH (350 m for winter and 450 m for spring) and no-precipitation. As shown in Fig. 3, persistent air stagnation conditions can lead to air pollution episodes; a stagnation event was claimed to last at least 3 days in this study (the sensitivity of air stagnation event duration was discussed in the SI).

**Figure 5:**
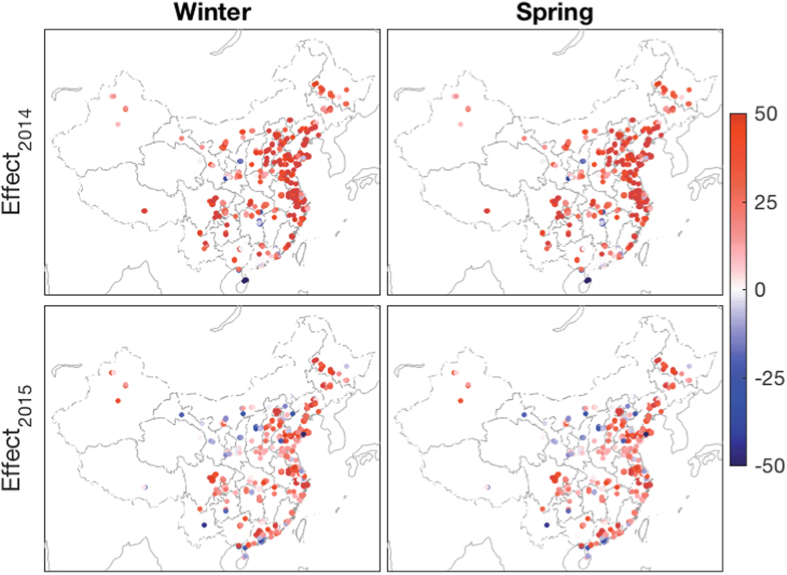
Air stagnation effect during the winter and spring of 2014 and 2015 (unit: %). Air stagnation effect was defined as the relative difference in PM_2.5_ concentration on air stagnation and no-stagnation days relative to the seasonal mean of the specific year. The majority of stations show a higher PM_2.5_ concentration during air stagnation events (positive effect). However, air stagnation at few stations over northern and northwestern China show a weak or even negative effect in spring, which could be attributed to the occurrence of sand storms in heavy winds. Statistical results are shown in Table 3. Results for air stagnation events, defined as the presence of an air stagnation day (at least one stagnation day), are shown in Fig. S1. This figure was produced by Matlab version 7.13 (http://www.mathworks.com/products).

Figure 5 shows the relative difference in PM_2.5_ concentrations between stagnation and no-stagnation periods, which considered as the effect of the air stagnation event on air pollutant accumulation. The effect of air stagnation on PM_2.5_ was greater in the high-pollution scenario, i.e., stronger in 2014 than in 2015, and greater in winter than in spring due to the more frequent severe pollution occurrences, as shown in Fig. 2. PM_2.5_ concentrations during stagnation events were 49% higher than during no-stagnation events during the winter of 2014 and 40% higher for the winter of 2015 (Table 3). Air stagnation effects were relatively weaker during the springtime, with a 26–32% higher PM_2.5_ concentration during stagnation events than that in no-stagnation period. The difference of PM_2.5_ concentrations between air stagnation and no-stagnation period was small and even reversed at some stations over northwestern China during the springtime, which may be due to the increased likelihood of sand and dust storms with heavy winds in the arid and semi-arid regions in no-stagnation events.

**Figure 6:**
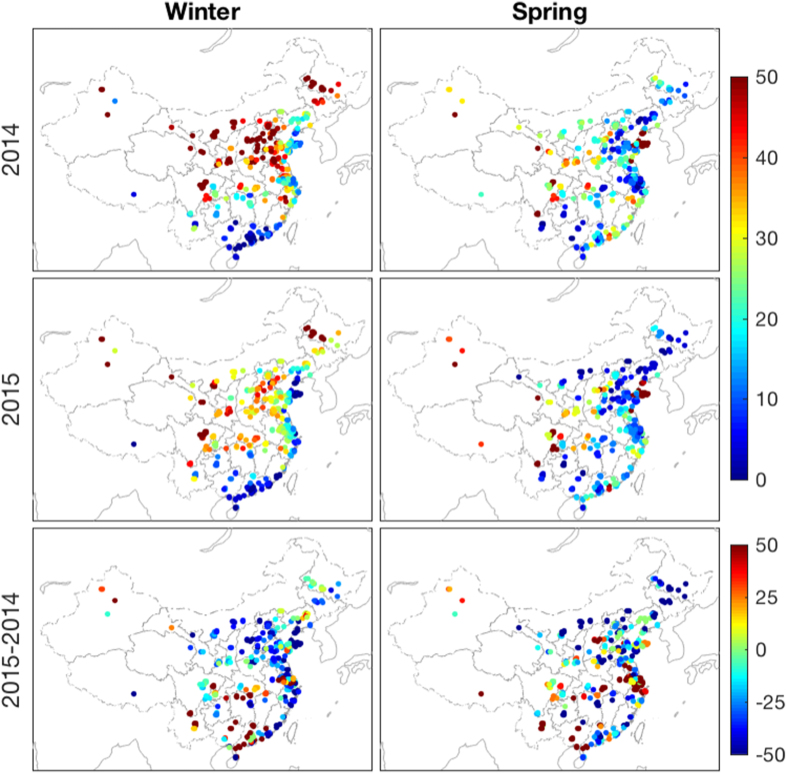
Occurrence of air stagnation events during winter and summer of 2014 and 2015 (top two panels) and the relative difference between these two years (bottom panel) (Unit: %). A total of 70% and 57% of the stations showed decreased air stagnation occurrences during the 2015 winter and spring, compared to that of 2014. The magnitudes of decreased frequency were much higher in winter than in spring. Statistical results are shown in Table 3. This figure was produced by Matlab version 7.13 (http://www.mathworks.com/products).

PM_2.5_ accumulates on air stagnation days and exacerbates local pollution. More frequent air stagnation leads to worsening air quality under similar emission conditions, i.e., lower air-stagnation frequency indicates better atmospheric diffusion ability. Figure 6 exhibits the occurrence frequency of air stagnation events during the study periods, i.e., the proportion of the total number of days during all the stagnation events in the available seasonal records. It shows more frequent air stagnation events during winter than spring. The higher frequency of air stagnation over inland China contrasts with the infrequent stagnations along the coastlines during winter, which may be due to a heavy land-sea breeze. The spatial difference in air stagnation occurrences was slight during the spring, with a relatively higher frequency over Xinjiang, Gansu and Sichuan provinces.

There were significantly fewer air stagnation events during the winter of 2015 than that of 2014, with 70% of the stations showing lower air stagnation frequency in the winter of 2015 (shown in Fig. 6 and Table 3). However, in spring, only 57% of the stations displayed a better atmospheric diffusion environment in 2015 compared with the previous year, and these stations were primarily located over northern and northeastern China. In addition, the magnitude of decreased air stagnation frequency was greater in winter than in spring (Fig. 5). The greater reduction of air stagnation occurrences over the majority of stations during the winter of 2015 may led to the relatively stronger improvement in air quality during winter than spring (20% decrease in PM_2.5_ for winter and 14% decrease for spring, as shown in Fig. 1).

We used the following method to quantify the contribution of changed atmospheric diffusion conditions to the improvement of air quality in China:


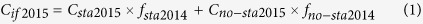


where *C*_*if2015*_ indicates the seasonal PM_2.5_ concentration at a specific station in 2015 if the air-stagnation frequency remained the same as in 2014. *C*_*sta2015*_ (*C*_*no-sta2015*_) indicates the seasonal mean PM_2.5_ concentrations during the stagnation or no-stagnation periods of 2015, respectively. *f*_*sta2014*_ (*f*_*no-sta2014*_) is the seasonal occurrence frequency of air stagnation or no-stagnation events in 2014. Seasonal concentrations and frequencies during winter and spring were used separately.

We compared the relative difference of *C*_*if2015*_ to the actual observations in 2015 to estimate the variation of PM_2.5_ due to the different atmospheric diffusion condition in 2015:


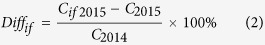


where *Diff*_*if*_ is the relative difference of *C*_*if2015*_ to the actual observations in 2015, and *C*_*2014*_ (*C*_*2015*_) is the actual observed seasonal mean PM_2.5_ concentration in 2014 or 2015:


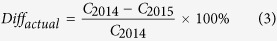


*Diff*_*actual*_ indicates the actual observed difference between 2014 and 2015. *Diff*_*if*_*/Diff*_*actual*_ shows the contribution of atmospheric conditions to the decreased PM_2.5_ concentration in 2015.

**Figure 7:**
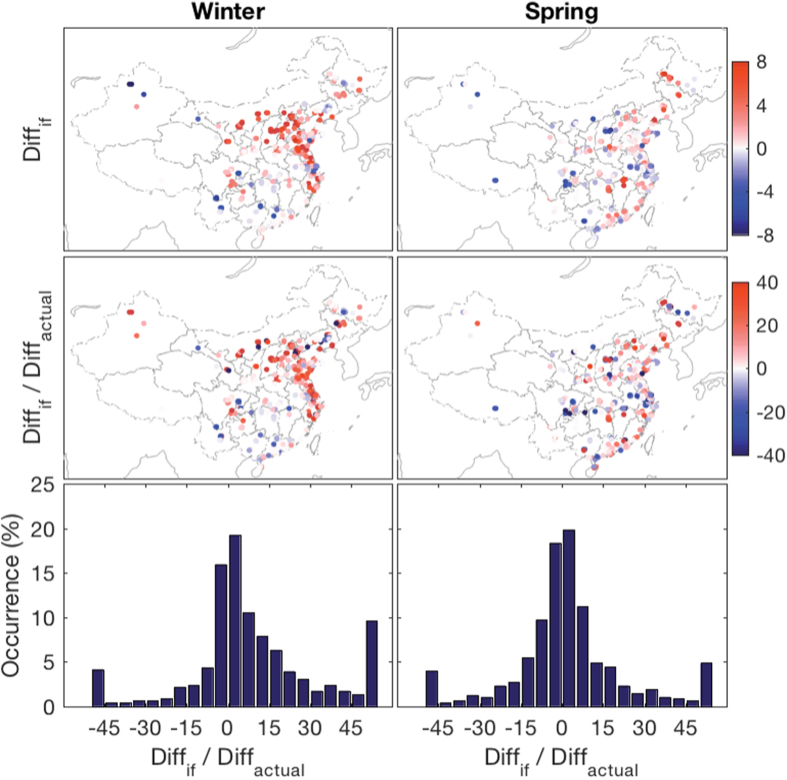
Relative difference between PM_2.5_ concentrations in 2015 but based on the 2014 air stagnation frequency and actual observed PM_2.5_ concentration in 2015 (top panel, unit: %), contribution of improved atmospheric diffusion conditions to the decreased PM_2.5_ in 2015 (middle panel, unit: %) and its probability distribution function (bottom panel). Positive *Diff*_*if*_ indicates that the air quality of 2015 would be worse than the actual observed quality if the atmospheric conditions were the same as in 2014. The favorable atmospheric diffusion conditions in the winter of 2015 accounted for approximately 40% of the total decreased PM_2.5_ concentration over central and northern China. The overall averaged contribution of atmospheric conditions in spring was slight. Results for air stagnation events defined as at least one stagnation day are shown in Fig. S3. This figure was produced by Matlab version 7.13 (http://www.mathworks.com/products).

Figure 7 shows the relative difference in PM_2.5_ concentrations between those actually observed during 2015 and those if all the stations had the same atmospheric conditions as in 2014. During the winter, 68% of the stations would have worse air quality levels than the observed level, which indicates that atmospheric conditions in 2015 were more favorable for pollutant removal; however, the proportion of stations decreased to 53% during the spring. 40% of the decreased PM_2.5_ in 2015 winter over Hebei, Henan, Shandong and Shanxi provinces can be attributed to the better atmospheric diffusion conditions in 2015 compared to 2014. For southern China, favorable atmospheric conditions in the winter of 2015 contributed to approximately 5–10% of the total decrease of PM_2.5_ concentration. The atmospheric diffusion ability of the 2015 spring was not as favorable as in winter, although approximately half of the stations had better air conditions than in the same period of the previous year (Fig. 6). Overall, the average contribution of atmospheric conditions was slight throughout the entire country.

## Conclusions and Discussion

China’s overall air quality significantly improved during the winter and spring of 2015 compared with that of 2014, with the near-surface PM_2.5_ concentrations decreased by 20% and 14%, respectively. The occurrences of very unhealthy and hazardous air quality decreased by 11%, which corresponds to a 6% more frequent normal unhealthy conditions in the winter of 2015 compared with 2014. In the spring of 2015, good and moderate air-quality days occurred 11% more frequently than in the same period of the previous year, with a corresponding infrequency of unhealthy conditions.

A seasonal dependence of PM_2.5_ concentrations on surface winds, boundary layer height and the occurrence of precipitation was established and used to define the presence of air stagnation conditions. Air stagnation events were defined as comprising at least 3 continuous air stagnation days. It showed higher PM_2.5_ concentrations during stagnation periods compared with no-stagnation events, particularly in the winter of 2014, with 49% higher PM_2.5_ concentration during air stagnation periods. Lower frequency of air stagnation events occurred at 70% and 57% of stations during the 2015 winter and spring, respectively, compared with the same periods of the previous year. Compared with the spring of 2015, the more significant improvement in air quality during the winter because the reduced air stagnation event frequency was observed at more stations in winter of 2015. The favorable atmospheric diffusion conditions contributed to approximately 40% of the total decreased concentrations during the winter of 2015 compared with the same period of the previous year over central and northern China (Hebei, Henan, Shandong and Shanxi Provinces). The atmospheric diffusion ability during the spring of 2015 was not as favorable as in the winter, and the average contribution of atmospheric conditions was slight throughout the country.

**Figure 8:**
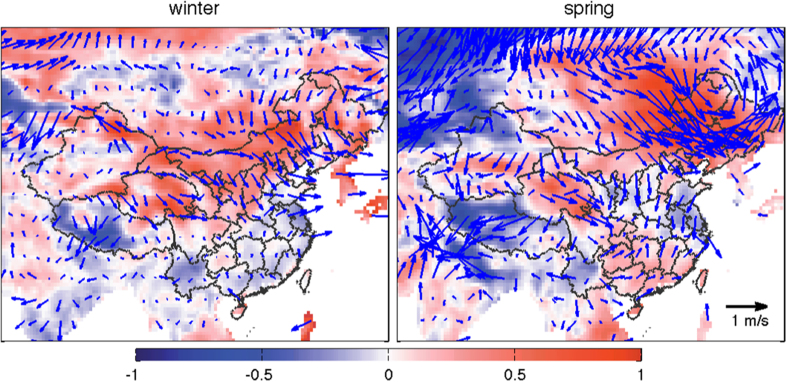
Anomalies of wind field between 2014 and 2015 (2015 minus 2014; wind speed anomaly is shaded). Compared with the previous year, stronger northerly wind occurred over northern and northeastern China in winter and spring 2015, respectively, during which the occurrence of air stagnation events decreased. This figure was produced by Matlab version 7.13 (http://www.mathworks.com/products).

Significant northerly wind anomalies occurred over northern China in 2015 compared with the previous year (as shown in Fig. 8), providing cleaner air and strengthening the horizontal transport of highly polluted regions. The stronger and northerly wind field anomalies could be attributed to the strong El Niño that developed in 2015^13^,^48^.

The average PM_2.5_ concentrations under air stagnation and no-stagnation conditions worked with the occurrence frequency of air stagnation and no-stagnation conditions were used to estimate the PM_2.5_ concentration in 2015 if it has the same atmospheric diffusion conditions as 2014 in Eq. (1). The PM_2.5_ concentrations were assumed to be the same on all air stagnation (or no-stagnation) days in our method. Actually, the air pollutant concentrations are significantly different on all available air stagnation (or no-stagnation) days, especially in different evolution stage of an air pollution episode^39^, which will increase the uncertainty of our estimation about the PM_2.5_ concentration in 2015.

This study only considered the physical removal of meteorological processes; however, the specific atmospheric conditions in a similar atmospheric diffusion condition could be different (e.g., temperature and humidity) which can effect certain chemical reactions during the formation of secondary aerosol^1^,^22^,^49^,^50^. In addition, high concentrations of surface air pollutants can locally reduce the amount of solar radiation reaching the earth’s surface; thus suppressing the development of an atmospheric boundary layer. The consequent shallow boundary layer height increases surface PM_2.5_ concentrations by compressing their diffusion volume^51^,^52^. Moreover, the increase in absorptive aerosols can enhance the atmospheric stability, thus impairing the downward transport of momentum and leading to worsened diffusion conditions with low-velocity surface winds^53^,^54^. The contribution of meteorological conditions to the chemical reaction of aerosol and the feedback between air pollutants and boundary layer processes are complicated and require further exploration.

## Data and Method

### Data

The real-time hourly PM_2.5_ concentration dataset has been continuously recorded by the Chinese Ministry of Environmental Protection and made publicly accessible since January 2013. The mass concentrations of PM_2.5_ are measured using either the micro-oscillating balance method or the β absorption method with commercial instruments. The instrumental operation, maintenance, data assurance and quality control are conducted according to HJ 655–201 Standards^5^. Some meteorological variables involved in this study (i.e., boundary layer height and surface wind) were obtained from a 0.25°*0.25° daily four-time ERA-Interim reanalysis dataset. Precipitation events were defined as the presence of rain, hail or snow rather than the daily precipitation amounts because of its high number of missing values over China, based on the US National Climate Data Center (NCDC) Global Surface Summary of the Day (GSOD) database.

### Method to quantify the criterion of air stagnation condition

Figure 3 shows that the sustained weak surface wind and a shallow boundary layer can trigger an air pollution episode, which can be broken by heavy surface wind or a well-developed boundary layer. Daily surface wind speed and boundary layer height were used to indicate the horizontal and vertical diffusion ability of the atmosphere. Daily PM_2.5_ concentrations were normalized by the monthly mean of the specific year to avoid the effects of spatial and temporal variation. All of the precipitation events were excluded in the dependence relationship to rule out the effect of wet deposition, i.e., the air stagnation condition will be terminated only if it rains. In general, near-surface PM_2.5_ concentrations decreased with the increase of surface wind and BLH. A shallow boundary layer (350 m for winter and 450 m for spring) restricts the diffusion of near-surface pollutants. And weak surface wind (2.7 ms^−1^ for winter and 3 ms^−1^ for spring) is favorable for the accumulation of air pollutants; however, this stagnation condition can be disrupted by a well-developed boundary layer during the winter. Atmospheric conditions in which the average daily normalized PM_2.5_ concentrations exceeded 100% were considered as air stagnation conditions, as shown in the lower-left corner of the dashed line in Fig. 4. All rainy days were defined as no-stagnation days because of the wet deposition affect.

A day can be designated as a stagnation day or a no-stagnation day based on its specific surface wind, BLH and the presence of precipitation. A stagnation event was claimed to last at least 3 days in this study, and the differences of PM_2.5_ concentrations between air stagnation and no-stagnation events were compared to estimate the effect of air stagnation event on air pollutant diffusion. Sensitivity of air stagnation event duration was discussed in the SI, which indicated that the one-day air stagnation showed the same pattern with that of three-day air stagnation events, but with a small magnitude of impact on air pollutant dispersion.

## Additional Information

**How to cite this article**: Wang, X. *et al*. Contribution of Atmospheric Diffusion Conditions to the Recent Improvement in Air Quality in China. *Sci. Rep.* **6**, 36404; doi: 10.1038/srep36404 (2016).

**Publisher’s note:** Springer Nature remains neutral with regard to jurisdictional claims in published maps and institutional affiliations.

## Supplementary Material

Supplementary Information

## Figures and Tables

**Table 1:** Seasonal mean PM_2.5_ concentrations during winter and spring of 2014 and 2015 and the relative difference between these two years.

	2014 (μgm^−3^)	2015 (μgm^−3^)	Difference (%)
Winter	99.8	76.8	−20.05
Spring	58.8	49.5	−14.05

**Table 2:** Comparison of the occurrence of different air-quality levels between 2014 and 2015.

AQI level	Good + moderate	Unhealthy for sensitive + unhealthy	Vary unhealthy + hazardous
*PM*_2.5_ concentration (μgm^−3^)	<35.4	35.5–150.0	>150.5
Winter (%)	3.95	6.17	−11.3
Spring (%)	10.6	−9.26	−2.3

A positive value indicates a more frequent occurrence of the specific air quality in 2015 than in 2014 (detailed thresholds of each air quality level were summarized in Table S1).

**Table 3:** Air stagnation effect and occurrence frequency during 2014 and 2015 and the relative difference in air stagnation frequency between the two years.

		2014 (%)	2015 (%)	Difference (%)
Air stagnation effect	Winter	40.34	34.74	\\
	Spring	21.87	27.83	\\
Air stagnation frequency	Winter	31.15	24.95	−17.35 (73.05%)
	Spring	19.14	17.56	−15.35 (62.30%)

The value in the brackets of the last column indicates the percentage of stations with decreased air-stagnation frequency in 2015 compared with the same period in the previous year.
