# Supplementary material for: Contribution of Atmospheric Diffusion Conditions to the Recent Improvement in Air Quality in China
Source: Sci Rep. 2016 Nov 2;6:36404. doi: 10.1038/srep36404 (PMC5090992; doi:10.1038/srep36404)
Supplement: Supplementary Information [file srep36404-s1.pdf]

Contribution of Atmospheric Diffusion Conditions to the Recent Improvement in Air Quality in China

Xiaoyan Wang[1,2,3], Kaicun Wang[2,3*], Liangyuan Su[2,3]

[1]Insitude of Atmospheric Science, Fudan University, Shanghai, 200433, China

[2]College of Global Change and Earth System Science, Beijing Normal University, Beijing, 100875, China

[3]Joint Center for Global Change Studies, Beijing, 100875, China

Table S1. Air quality index (AQI) categories and corresponding $PM_{2.5}$ concentration thresholds. Concentration thresholds are calculated according to the method of the US EPA by taking $PM_{2.5}$ as the primary pollutant.

Levels of Health Concern	Air Quality Index (AQI)	$PM_{2.5}$ (μg/m^3)
Good	0-50	0-12
Moderate	51-100	12.1-35.4
Unhealthy for sensitive groups	101-150	35.5-55.4
Unhealthy	151-200	55.5-150.4
Very Unhealthy	201-300	150.5-250.4
Hazardous	301-500	>250.5

Table S2. One-day air stagnation effect and frequency during 2014 and 2015 (statistical results of Fig. S1-2). A one-day air stagnation event was defined to last at least one day rather than 3 days. The value in the brackets in the last column indicates the percentage of stations with decreased air stagnation frequency in 2015 compared with the same period in 2014.

		2014 (%)	2015 (%)	Difference (%)
Air stagnation effect	Winter	40.68	34.12	\\
	Spring	21.61	23.25	\\
Air stagnation frequency	Winter	42.26	39.51	-5.05 (68.95%)
	Spring	35.40	34.36	-1.53 (45.29%)

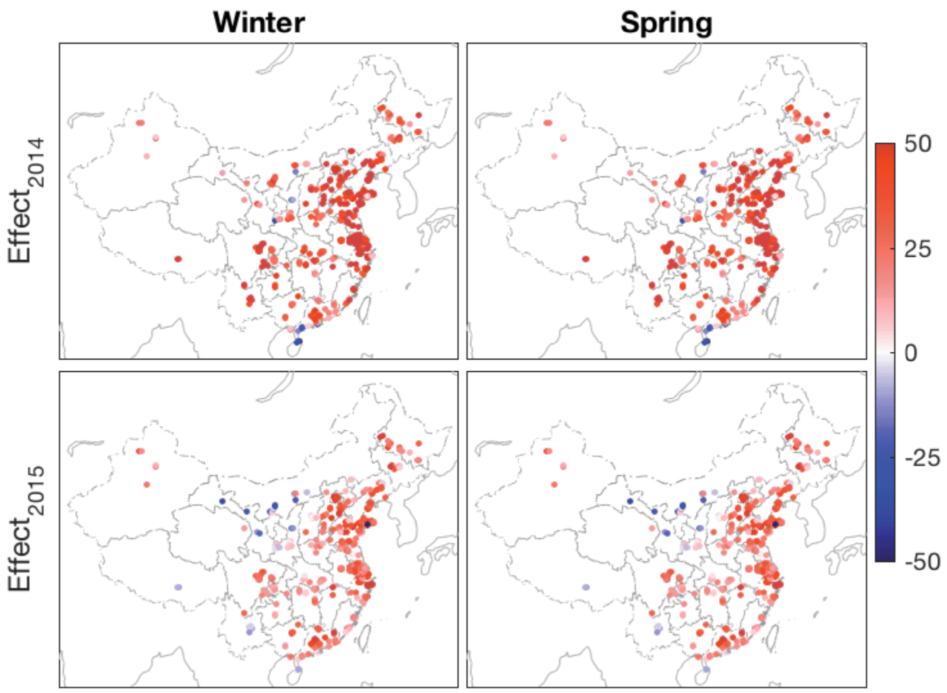

Figure S1. Air stagnation effect during the winter and spring of 2014 and 2015 (unit: %). The air stagnation effect was defined as the relative $PM_{2.5}$ difference between air stagnation days and no-stagnation days (see Fig. 4), but the stagnation event lasted at least one day rather than 3 days. The effect of one-day air stagnation events was relatively weaker than that of 3-day events. Statistical results of this figure are summarized in Table S2. This figure was produced by Matlab version 7.13 (http://www.mathworks.com/products).

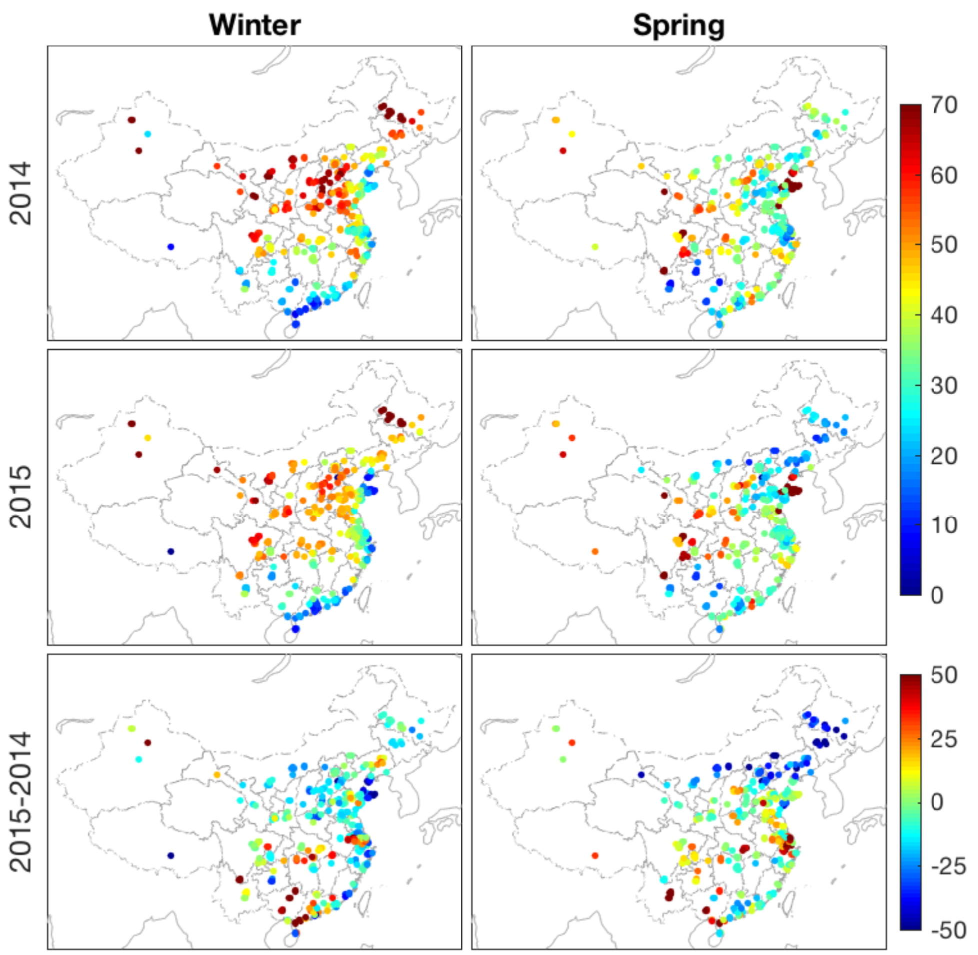

Figure S2. Occurrence of one-day air stagnation events during the winter and summer of 2014 and 2015 (left two panels) and the relative difference between the two years (right panel) (unit: %). The frequency of one-day air stagnation events was higher than that of 3-day stagnation events because short-term (less than 3 days) stagnation events were excluded in 3-day cases. Statistical results of this figure are summarized in Table S2. This figure was produced by Matlab version 7.13 (http://www.mathworks.com/products).

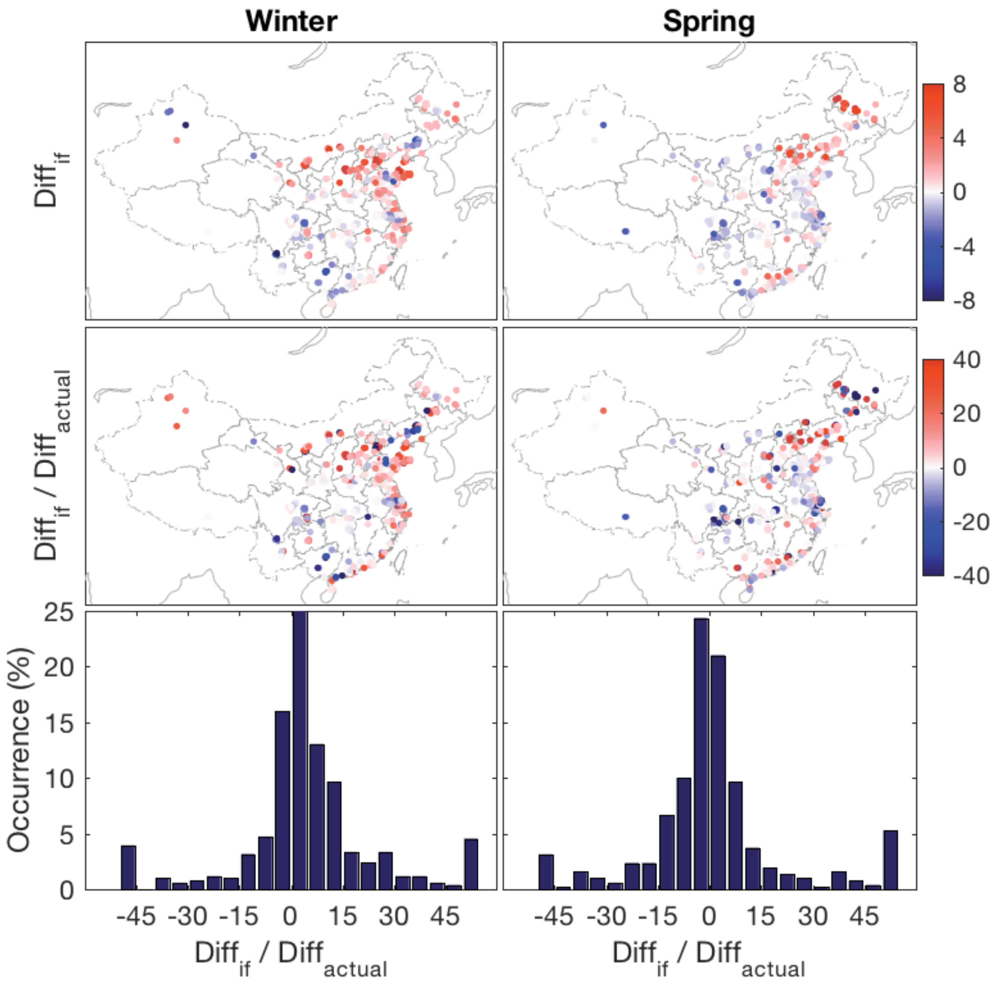

Figure S3. Relative difference in 2015 $PM_{2.5}$ concentration between the actual observations and the 2014 one-day air stagnation frequency (top panel, unit: %), the contribution of improved atmospheric diffusion conditions to the decreased $PM_{2.5}$ in 2015 (middle panel, unit: %) and the occurrence of the contribution (bottom panel). The same as Fig. 6. This figure was produced by Matlab version 7.13 (http://www.mathworks.com/products).
